# Bone metastases in soft tissue sarcoma: a survey of natural history, prognostic value and treatment options

**DOI:** 10.1186/2045-3329-3-6

**Published:** 2013-04-17

**Authors:** Bruno Vincenzi, Anna Maria Frezza, Gaia Schiavon, Daniele Santini, Palma Dileo, Marianna Silletta, Delia Delisi, Francesco Bertoldo, Giuseppe Badalamenti, Giacomo Giulio Baldi, Stefania Zovato, Rossana Berardi, Marco Tucci, Franco Silvestris, Angelo Paolo Dei Tos, Roberto Tirabosco, Jeremy Simon Whelan, Giuseppe Tonini

**Affiliations:** 1 Department of Medical Oncology, Universita’ Campus Bio-Medico, Rome, Italy; 2 Breast Unit, Royal Marsden Hospital, London, UK; 3 Department of Oncology, London Sarcoma Service, University College Hospital, London, UK; 4 Department of Medicine, Universita’ di Verona, Verona, Italy; 5 Department of Medical Oncology, Policlinico P. Giaccone, Palemo, Italy; 6 Department of Medical Oncology, Ospedale Santa Chiara, Pisa, Italy; 7 Department of Medical Oncology, Istituto Oncologico Veneto, Padova, Italy; 8 Department of Medical Oncology, Universita’ Politecnica delle Marche, Ancona, Italy; 9 Department of Biomedical Sciences and Medical Oncology, University of Bari ‘Aldo Moro’, Bari, Italy; 10 Department of Pathology, Royal National Orthopaedic Hospital, London, UK; 11 Department of Medical Oncology, Daniel den Hoed Cancer Center, Erasmus University Medical Center, Rotterdam, The Netherlands; 12 Department of Pathology, General Hospital, Treviso, Italy

**Keywords:** Bone metastases, Soft tissue sarcoma, Skeletal related events, Biphosphonate

## Abstract

**Background:**

We surveyed the natural history of bone metastases in patients affected by soft tissue sarcoma (STS).

**Methods:**

This multicenter retrospective observational study included 135 patients. Histological subtype, characteristics of bone metastases, treatment, skeletal related events (SREs) and disease outcome were recorded.

**Results:**

The most represented histological subtypes were leiomyosarcoma (27%) angiosarcoma (13%) and undifferentiated sarcoma (8%). Axial skeleton was the most common site for bone involvement (70%). In 27% of cases, bone metastases were present at the time of diagnosis. Fifty-four (40%) patients developed SREs and the median time to first SRE was 4 months (range 1–9). The most common SRE was the need for radiotherapy (28%) followed by pathological fracture (22%). Median survival after bone progression was 6 months (range 1–14). SREs were associated with decreased overall survival (OS) (P = 0.04). A subgroup analysis revealed that bisphosphonates significantly prolonged median time to first SRE (5 versus 2 months; P = 0.002) while they did not determine an improvement in OS, although a favourable trend was identified (median: 7 versus 5 months; P = 0.105).

**Conclusions:**

This study illustrates the burden of bone disease from STS and supports the use of bisphosphonates in this setting.

## Introduction

Soft tissue sarcomas (STS) are a group of rare and heterogeneous cancers, accounting for less than 1% of all human tumors. Their biological behavior is characterized by local aggressiveness and tendency to hematogenous spreading. Almost 50% of STS patients develop metastatic disease [1], mainly within three years from initial diagnosis. The distribution of metastases from STS varies, depending on the primary site and histological subtype. For example, those arising from the extremities mainly spread to the lungs, and those arising from the abdominal cavity, usually metastasizing to liver and peritoneum.

Even if no extensive data are currently available on the skeletal involvement in STS patients, the daily clinical practice suggests that the development of bone metastases and the subsequent occurrence of skeletal related events (SREs) (namely pathological fracture, hypercalcaemia, spinal cord compression, need for surgery or palliative radiotherapy for refractory pain) [2] can be part of the natural history of this disease, can significantly modified a patient’s performance status and deeply affect quality of life. Furthermore, the role of bisphosphonates therapy, known to be effective in preventing or delaying SREs and in relieving from bone pain in different human tumors [3,4], has never been assessed in STS patients.

The aim of this retrospective multicenter observational study was to better define the natural history of skeletal metastases from STS, to investigate their possible prognostic value and to explore different treatment options which might guide future practice.

## Patients and methods

### Patient population

Almost 1250 patients affected by metastatic soft tissue sarcoma have been screened. Among these, 135 patients with radiologically (plain films, CT scan, MRI scan) proven bone metastases from STS have been selected and retrospectively included in the present analysis. All patients were diagnosed and treated between 1999 and 2011 at University College Hospital (London Sarcoma Service), University Campus Bio-Medico (Rome), Ospedale Santa Chiara (Pisa), Policlinico P. Giaccone (Palemo), Istituto Oncologico Veneto (Padova), Universita’ Politecnica delle Marche (Ancona), and Ospedale Giovanni XXIII (Bari).

### Data collection

The following clinical data have been retrospectively collected: age, gender, primary histological subtype (classified according to 2002 World Health Organization classification of the tumors [5]), grade (according to the French federation of Cancer Centre Sarcoma Group [6]), primary site, presence of bone metastases at the time of diagnosis, number (equal or less than 5, more than 5) and site of bone metastases, treatments received for bone lesions (chemotherapy, zoledronic acid, palliative radiotherapy or surgery), presence of SREs, time to first SREs and survival from the time of skeletal disease diagnosis. The clinical data were both manually and electronically extracted. The completeness of the series of each centre was based on the contribution of each author. The study has been conducted in compliance with the Helsinki Declaration and has been approved by the ethic committee at University Campus Bio-Medico.

### Statistical analysis

In the univariate model all the clinical variables were evaluated as predictors for shorter time to bone metastasis, higher risk of skeletal morbidity (SREs), and shorter time from SREs to death. Patients who did not have a recorded date for a specific event were censored after the date of last follow-up. All survival intervals were determined by the Kaplan-Meier product-limit method [7] while differences in survival according to clinical parameters or treatment were evaluated by the log-rank test [8] and described by the Kaplan-Meier method. The last follow-up for patients included in the analysis was November 2011 and the event was death. SPSS software (version 17.00, SPSS, Chicago) was used for statistical analysis. A P value of less than .05 indicated statistical significance.

## Results

### Patient population

Among the enrolled 135 patients 53% were male (71/135). The median age was 52 years (range 23 to 86). All patients were affected by a histologically proven sarcoma of the soft tissue and all the diagnoses were centrally reviewed by a pathologist with expertise in this field. Leiomyosarcoma was diagnosed in 27% (36/135) of the patients while 14% (19/135) were affected by undifferentiated pleomorphic sarcoma, 13% (17/135) by angiosarcoma, 7% (10/135) by liposarcoma, 7% (9/135) by malignant epithelioid hemangioendothelioma, 6% (8/135) by malignant peripheral nerve sheet tumor (MPNST), 5% (7/135) by synovial sarcoma and 21% (29/135) by other different histological subtypes. The histological grade was G1 in 4% (6/135) of patients, G2 (28/135) in 21% of patients, and G3 in 75% (101/135). The primary site of the disease was limbs or limb girdles in 50% (67/135) of patients, body cavity in 33% (45/135) and trunk in 17% (23/135). Patient characteristics are summarized in Table 1.

**Table 1:** Patient characteristics

**Characteristic**	**N patients (%)**
**Median age (years)**	52 (range: 23–86)
**Gender**	
• Male	71 (53)
• Female	64 (47)
**Histology**	
• Leiomyosarcoma	36 (27)
• Undifferentiated pleomorphic sarcoma	19 (14)
• Angiosarcoma	17 (13)
• Liposarcoma	10 (7)
• Malignant epithelioid hemangioendothelioma	9 (7)
• MPNST	8 (6)
• Synovial sarcoma	7 (5)
• Others	29 (21)
**Tumour grade**	
• G1	6 (4)
• G2	21 (28)
• G3	101 (75)
**Primary site**	
• Limbs or limb girdles	67 (50)
• Body cavity	45 (33)
• Trunk	23 (17)

### Natural history of bone metastases from soft tissue sarcoma

In 27% (36/135) of cases, bone metastases were present at the time of diagnosis. Less than 5 bone metastases were detected in 60% (81/135) of patients while more than 5 were present in the remaining 40% (54/135). Among the analyzed patients, the spine was involved in 51% (69/135) of cases, being the most common skeletal site; hip/pelvis was involved in 20% (27/135) of patients, long bones in 15% (20/135) and other sites 14% (19/135). A SRE developed in 40% (54/135) of patients and median time to first SRE was 4 months (range: 1–9). The most common SRE was the need for radiotherapy occurring in 28% (38/135) of patients followed by pathologic fracture (22%, 30/135), need for surgery (21%, 29/135), cord compression (13%, 18/135) and hypercalcaemia occurring only in 3% (4/135) of patients. These results are summarized in Table 2.

**Table 2:** Natural history of bone metastases from soft tissue sarcoma

	**N patients (%)**
**Bone metastases onset**	
• Time of diagosis	36 (27)
• After diagnosis	99 (73)
**Number of bone metastases**	
• ≤ 5	81 (60)
• > 5	54 (40)
**Bone metastases site**	
• Spine	69 (51)
• Hip/pelvis	27 (20)
• Long bones	20 (15)
• Others	19 (14)
**SRE development**	
• Yes	54 (40)
• No	81 (60)
• Median time to first SRE (months)	4 (range: 1–9)
**SRE type**	
• Palliative radiotherapy	28 (38)
• Pathological fracture	30 (22)
• Cord compression	18 (13)
• Hypercalcaemia	4 (3)
• Palliative surgery	29 (21)

### Prognostic value of bone metastases and treatment options

Patients survived for a median of 6 months after diagnosis of bone metastases (range: 1–14). The number of bone metastases did not significantly correlate with survival in STS patients (P = 0.63), while both the occurrence of SREs and an early time to first SRE were associated with a decreased overall survival (OS) (P = 0.04 and P = 0.002).

Among the enrolled patients, 72% (97/135) received chemotherapy. The median number of treatment lines was 2 (range: 0–4) and the most wildly used drugs included doxorubicin, ifosfamide, gemcitabine, docetaxel and paclitaxel. Only 60% (81/135) were started on bisphosphonates at the time of bone metastasis diagnosis. Radiotherapy was given to 31% (42/135) of the patients, 21% (29/135) were treated with surgery and 2% (3/135) with other local approaches. On the contrary, 39% (52/135) did not receive any specific treatment. These results were summarized in Table 3. A subgroup analysis revealed that bisphosphonates significantly prolonged median time to first SRE (5 versus 2 months; P = 0.002). Conversely, bisphosphonate therapy did not determine an improvement in terms of OS, even if a favorable trend was identified for treated patients (median, 7 versus 5 months, respectively; P = 0.105). These results are summarized in Table 4.

**Table 3:** Treatment options for bone metastases from soft tissue sarcoma

**Treatment**	**N patients (%)**
• Biphosphonates	81 (60)
• Radiotherapy	42 (31)
• Surgery	29 (21)
• Other	3 (2)
• No specific treatment	52 (39)

**Table 4:** Impact of bisphosphonate therapy on overall survival and time to first skeletal related events

	**Bisphosphonate**	**No bisphosphonate**	**P value**
Median overall survival (months)	7	5	0.105
Median time to first SRE (months)	5	2	0.002

## Discussion

The skeleton is the third most frequent site of metastases from solid tumors and the vast majority of bone metastases affect patients with primary breast, lung or prostate cancer. However, the development of metastatic bone disease and the occurrence of SREs can complicate several different malignancies, causing a worsening of patient’s quality of life and requiring a complex management which significantly impact on health resources [9].

Among patients affected by STSs, bone metastases occur in almost 10%, with a great variability depending on the histological subtypes. From previous smaller reports [10,11], angiosarcoma, dedifferentiated liposarcoma and alveolar soft part sarcoma showed the higher incidence of skeletal metastases with a percentage of bone involvement of almost 50%. The axial skeleton is the most common site of localization while the involvement of long bones is rarer and usually limited to diaphyses. Radiologically, bone metastases from STS are described as lytic in more than 80% of cases [12] and the detection of a pathological fracture by radiography is not an uncommon finding.

Although our dataset confirmed the variability in bone metastases incidence among different subtypes of sarcoma, leiomyosarcoma, undifferentiated pleomorphic sarcoma and angiosarcoma were the most common histotypes associated with bone involvement. As expected, high histological grade and a primary tumor arising from limbs or limb girdles were both found to be risk factors for the development of bone metastases. Concordantly with previous findings, the axial skeleton was involved in 70% of the enrolled patients while long bones only in 15%. Moreover, for the first time in the present study, the rate of SREs was assessed and they have been found to occur in 40% of the included patients. The most common SREs were represented by the need for radiotherapy, mainly in the context of pain management, and pathological fractures. Interestingly, as already known for breast [13] and prostate [14] cancer patients, the occurrence of SREs, especially within six months from the diagnosis of bone metastases, strongly correlates with a decreased OS (P = 0.002).

Despite these results, which clearly describe the extent of this problem and its relevance in terms of prognosis and quality of life, there are currently no clear guidelines regarding the management of bone metastases from STS. This issue is confirmed by the extreme heterogeneity of treatment approaches observed in this population. Radiotherapy was used in almost one third of the patients in our dataset, mainly to improve pain control or to reduce the risk of a pathological fracture, while the use of surgery was limited to 21% of the cases.

It is interesting to underline that 60% of the patients were started on bisphosphonates at the time of bone metastasis diagnosis, including pamidronate or zoledronic acid. The selection criteria for administration of bisphosphonates are not clear but are mainly dependent on institutional practice. The results from the present analysis show how the use of bisphosphonates in patients with bone metastases from STS significantly delays the first SRE (P = 0.002) and seems to be associated with a favorable trend in terms of survival, although the statistical significance was not reached (P = 0.105) in this cohort.

## Conclusions

On the basis of the present study, the need for a standardization of bone metastases treatment in STS patients is recommended in order to improve quality of life and perhaps impact on survival.

Although the value of our results is limited by the retrospective nature of the study we believe that, given the delay demonstrated in the time to the first SRE, the use of bisphosphonates in this setting should be considered.

Further studies are warranted which aim to corroborate these results and to properly explore the impact of bisphosphonates therapy on survival of STS patients.

## Abbreviations

STS: Soft tissue sarcoma; SREs: Skeletal related events; MPNST: Malignant peripheral nerve sheet tumor; OS: Overall survival.

## Competing interests

The authors have no competing interests to declare.

## Authors’ contributions

BV, GT, DS, AMF conceived the study and the design. AMF, GB, GGB, SZ, RB, MT, RT and FB carried out data collection and BV performed statistical analysis. AMF, GS, FS, APD, MS, PD, DD and JW helped to draft the manuscript. All authors read and approved the final manuscript.
